# DNC4mC-Deep: Identification and Analysis of DNA N4-Methylcytosine Sites Based on Different Encoding Schemes By Using Deep Learning

**DOI:** 10.3390/cells9081756

**Published:** 2020-07-22

**Authors:** Abdul Wahab, Omid Mahmoudi, Jeehong Kim, Kil To Chong

**Affiliations:** 1 Department of Electronics and Information Engineering, Jeonbuk National University, Jeonju 54896, Korea; me.wahabqayyum@gmail.com (A.W.); omidmahmoudi75@jbnu.ac.kr (O.M.); 2 Department of New & Renewable Energy, VISION College of Jeonju, Jeonju 55069, Korea; 3 Department of Electronics Engineering, Jeonbuk National University, Jeonju 54896, Korea; 4 Advance Electronics & Information Research Center, Jeonbuk National University, Jeonju 54896, Korea

**Keywords:** N4-methylcytosine, rosaceae genome, DNA encoding methods, computational biology, deep learning, bioinformatics

## Abstract

N4-methylcytosine as one kind of modification of DNA has a critical role which alters genetic performance such as protein interactions, conformation, stability in DNA as well as the regulation of gene expression same cell developmental and genomic imprinting. Some different 4mC site identifiers have been proposed for various species. Herein, we proposed a computational model, DNC4mC-Deep, including six encoding techniques plus a deep learning model to predict 4mC sites in the genome of *F. vesca*, *R. chinensis*, and Cross-species dataset. It was demonstrated by the 10-fold cross-validation test to get superior performance. The DNC4mC-Deep obtained 0.829 and 0.929 of MCC on *F. vesca* and *R. chinensis* training dataset, respectively, and 0.814 on cross-species. This means the proposed method outperforms the state-of-the-art predictors at least 0.284 and 0.265 on *F. vesca* and *R. chinensis* training dataset in turn. Furthermore, the DNC4mC-Deep achieved 0.635 and 0.565 of MCC on *F. vesca* and *R. chinensis* independent dataset, respectively, and 0.562 on cross-species which shows it can achieve the best performance to predict 4mC sites as compared to the state-of-the-art predictor.

## 1. Introduction

Dynamic DNA modifications, such as methylation and demethylation have an essential role in the regulation of gene expression. DNA methylation as a heritable epigenetic marker is one type of chemical modification of DNA, which alters genetic performance without altering the DNA sequence [1,2]. Several researches have shown that it has the ability to change DNA protein interactions, DNA conformation, DNA stability, and chromatin structure. Meanwhile, it can regulate some different functions including cell developmental, genomic imprinting, and gene expressions [3,4]. N4-methylcytosine (4mC), 5-Methylcytosine (5mC), and N6-methyladenine (6mA) as three common methylations by specific methyltransferase enzymes occur in both prokaryotes and eukaryotes [5,6,7].

In prokaryotes, the host DNA from exogenous pathogenic DNA can be identified by 6mA and 4mC [8], and also 4mC regulates DNA replication and its errors [9,10]. Meanwhile, 4mC as part of a restriction-modification (R-M) system prevents restriction enzymes from degrading host DNA [11]. In eukaryotes, 5mC has a crucial role in transposon suppression, gene imprinting, and regulation. By high sensitivity techniques, 6mA and 4mC can only be detected in eukaryotes [12].

The 5mC, as the most well-explored and common type of cytosine methylation plays a significant role in several biological processes [13] and can be caused by cancer, diabetes, and also some neurological diseases [14,15,16]. The 4mC as effective methylation protects its own DNA from the restriction of enzyme-mediated degradation. It has an important role in controlling some various processes including cell cycle, gene expression levels, differentiating self and non-self-DNA, DNA replication, and correcting DNA replication errors [9,17].

Some extensive experimental studies have been performed to detect 4mC sites in the whole genome such as 4mC-Tet-assisted bisulfite sequencing, methylation-precise PCR, mass spectrometry, and Single-Molecule of Real-Time (SMRT) sequencing [18,19,20,21]. The aforementioned experimental approaches are laborious and expensive work when performing genome-wide testing. Therefore, it is necessary to develop a computational method for identifying 4mC sites.

Lately, several 4mC sites identifiers [22,23] have been proposed for different species such as *C. elegans*, *D. melanogaster*, *A. thaliana*, *E. coli*, *G. subterraneus*, *G. pickeringii*. The i4mC-ROSE [24] as the first computational tool for predicting 4mC sites within the *Rosaceae* genomes has been proposed to identify the 4mC sites in the genomes of *F. vesca* [25] and *R. chinensis* [26]. It generated six probabilistic scores by using six encoding methods; random forest (RF), algorithms with k-space spectral nucleotide composition (KSNC), electron-ion interaction pseudopotentials (EIIP), k-mer composition (Kmer), binary encoding (BE), dinucleotide physicochemical properties (DPCP), and trinucleotide physicochemical properties (TPCP) that cover various aspects of DNA sequence information. Then, those scores were combined with a linear regression model for enhanced prediction performance [24]. The 4mcDeep-CBI [27] as a deep learning framework has been proposed to predict the 4mC sites in an expanded dataset of *Caenorhabditis elegans* (*C. elegans*). 3-CNN and BLSTM were used to extract deep information and to obtain advanced features.

In this work, a novel predictor, DNC4mC-Deep, has been established for the identification of 4mC sites in the genome of *F. vesca*, *R. chinensis*, and cross-species which is newly prepared. The overall framework of our work summarized as; Firstly we used the six encoding techniques named 2Kmer [28], 3Kmer [29], binary encoding (BE) [30,31], nucleotide chemical property (NCP) [32], nucleotide chemical property, and nucleotide frequency (NCPNF) [32], and multivariate mutual information (MMI) [33,34]. Then, we made a deep learning model by using the Convolution Neural Network (CNN). We applied a grid search algorithm to obtain the optimal model with tuned hyperparameters. All six encoding schemes were input separately in the optimal selected model and recorded the results of each encoding scheme and used the K-fold cross-validation method with the value of K as 10. To evaluate and analyze the results of the model on each encoding scheme, we used the performance evaluation metrics. We also presented two different applications; the first one is silico mutagenesis [35] representation using heat maps, and the second is distinguishing the most significant portions of a sequence using saliency maps [36]. After getting the results from the model by all six different feature encoding methods, we ended up with that Dinucleotide composition (DNC) is outperforms from all six encoding schemes and the state-of-the-art model. In comparison to the state-of-the-art model, DNC4mC-Deep successfully achieves 0.635, 0.565, and 0.562 of MCC on *F. vesca*, *R. chinensis*, and cross-species independent dataset, respectively.

## 2. Materials and Methods

### 2.1. Benchmark Datasets

The benchmark dataset of DNA 4mC obtained from Md. Mehedi Hassan et al. [24]. It contains the *F. vesca* and *R. chinensis* genome. To prepare the high-quality dataset they have applied the sequences with ModQV score greater than 20, whereas the remaining sequences were excluded. To solve the homology bias problem, the CD-HIT-EST [37] software was used to exclude redundant sequences with a cut-off of 0.65. All sequences contain a central cytosine (C) nucleotide with a length of 41 base pairs (bp).

In both datasets, *F. vesca* and *R. chinensis* genome were considered 75% and 25% samples from all data as the training and the independent dataset. The training dataset consists of 4854 and 2337 positive DNA sequences as 4mC samples for *F. vesca*, and *R. chinensis*, severally. The negative DNA sequences, such as non-4mC, consists of 4854 and 2337 samples for *F. vesca*, and *R. chinensis* genome. Furthermore, the independent dataset included 1617 for both positive and negative DNA sequences of *F. vesca* genome whereas for *R. chinensis* positive and negative DNA contains 779 samples.

Moreover, we made the cross-species as a new benchmark dataset from the two above datasets. To avoid the redundancy in the original datasets we used CD-HIT-EST with different threshold values. The recent dataset was also divided into the training and the independent dataset with the same proportion (75% and 25% samples) where we obtained the cross-species dataset with the most attentive threshold at 0.80 containing 7190 and 5874 positive and negative DNA sequences, respectively, on the training dataset. Meanwhile, we assumed 2394 positive and 2234 negative DNA sequences on the independent dataset. The length of each sample is 41nt. Details of the benchmark datasets are shown in Table 1.

### 2.2. Feature Encoding Methods

Feature encoding has a vital role in the construction of the model [38]. A DNA sequence is represented as a fixed length of feature vectors which can be classified by deep learning algorithms. In this article, six various types of feature encoding methods, binary encoding [39], DNC (2kmer), TNC (3kmer) [40,41,42,43], Multivariate Mutual Information (MMI) [44], Nucleotide Chemical Property (NCP) and Nucleotide Chemical Property and Nucleotide Frequency (NCPNF) [28,29,45,46,47] were employed to formulate methylcytosine samples.

#### 2.2.1. Binary Encoding (BE)

Binary encoding is a simple and effective feature algorithm converts each nucleotide into a binary vector as follows: A (1, 0, 0, 0, 0), C (0, 1, 0, 0, 0), G (0, 0, 1, 0, 0), T (0, 0, 0, 1, 0) and N (0, 0, 0, 0, 0). A DNA sequence with *m* nucleotides can be represented into a vector of 5×m features [30,31].

#### 2.2.2. Kmer

Kmer is a common feature encoding algorithm that has been widely used in various prediction works [28,29,45,48,49]. A DNA sample is expressed as V = N1, N2, N3, … NL, where L denotes the length of the sequence and Ni is one of the regular nucleotides A, C, G, T, and N. In this work, di-nucleotide composition (DNC) and tri-nucleotide composition (TNC) were considered. In DNC all samples of 41 nt produce 40 components with the equation of L−k+1. The DNC scheme generated a 25 $\left(5^{2}\right)$ dimensional feature. Whereas in TNC samples of 41 nt generated 39 elements with the equation of L−k+2. The TNC form into a 125 ($5^{3}$)-dimensional vector. In both equations, the L denotes the length of the sequence and k represents the value of Kmer as an integer.

#### 2.2.3. Nucleotide Chemical Property (NCP)

The four nucleic acids have different chemical properties [50]. In terms of ring structures, A and G each contain two rings, whereas C and T contain only one. Regarding secondary structures, A and T form weak hydrogen bonds, whereas C and G form strong hydrogen bonds. In terms of chemical functionality, A and C can be classified into the amino group, while G and T can be classified into the keto group. The cluster of four nucleotides was shown in Table 2.

Three coordinates x, y, and z were used to represent ring structure, the hydrogen bond, the chemical functionality, respectively, and the value of 0 and 1 was assigned to each one. The feature extraction algorithm can be formulated as follows:

$$x_{i}=\{\begin{array}{cc} 1 & ifs_{i}\epsilon \left\{A,\;G\right\}\\ 0 & ifs_{i}\epsilon \left\{C,\;T\right\} \end{array},y_{i}=\{\begin{array}{cc} 1 & ifs_{i}\epsilon \left\{A,\;T\right\}\\ 0 & ifs_{i}\epsilon \left\{C,\;G\right\} \end{array},z_{i}=\{\begin{array}{cc} 1 & ifs_{i}\epsilon \left\{A,\;C\right\}\\ 0 & ifs_{i}\epsilon \left\{G,\;T\right\} \end{array}$$

where $n(s_{i})$ represents A, C, G, T, and N nucleotide, which can be converted by the coordinates (1, 1, 1), (0, 0, 1), (1, 0, 0), (0, 1, 0), and (0, 0, 0), respectively.

We also tried the nucleotide chemical properties (NCP) with the frequencies of each nucleotide (NF) position in a sample. The method was got from Chen et al. [32] for both encoding schemes. We integrated the NCP and NF to represent a matrix with 41 columns and 5 rows for each sample of the DNA sequence. Each DNA base of the sequence was designated as a column of the matrix and for each column, their initial three components were characterized as the nucleotide chemical property and the last one represented as a nucleotide frequency which we denoted as NCPNF.

#### 2.2.4. Multivariate Mutual Information (MMI)

MMI has been used in many works [33,34,51] to extract features of the nucleotides sequence. We used the MMI based feature encoding algorithm which was proposed by Pan et al. [52]. First of all, they modified a two-tuple and three-tuple nucleotides set as follows:

$$\begin{array}{cc} T_{2}= & \left\{AA,AC,AG,AT,AN,CC,CG,CT,CN,GG,GT,GN,TT,TN,NN\right\}\\ T_{3}= & \{AAA,AAC,AAG,AAT,AAN,ACC,ACG,ACT,ACN,AGG,AGT,\\ & AGN,ATT,ATN,ANN,CCC,CCG,CCT,CCN,CGG,CGT,CGN,\\ & CTT,CTN,CNN,GGG,GGT,GGN,GTT,GTN,TTT,TTN,NNN\} \end{array}$$

Then, the mutual information for the elements was calculated as a frequency of nucleotides in the sequence with respect to 2-tuple and 3-tuple. We extracted 55 MMI features.

## 3. The Proposed Deep Learning Model

In this study, an efficient deep learning model based on CNN was proposed for the identification of 4mC sites in the genome of *F. vesca*, *R. chinensis* and cross-species. CNN does not require manually extracted features like a conventional supervised learning processes. The immense advantage of a CNN, it can extract the features by itself automatically for the classification process. Additionally, a handy crafted feature can also be fed to CNN to build a heterogeneous model. A CNN has a big impact on various fields of natural language processing, image processing [53,54,55,56] and computational biology [57,58]. To get an optimum model we applied grid search and during learning the CNN, six hyperparameters were tuned. The ranges within each hyper-parameter was tuned to are listed in Table 3.

After getting the best model from the grid search, we used six different encoding schemes (DNC, TNC, BE, NCP, NCPNF, MMI) for the input of the CNN model. Each encoding technique converted into vectorization of the input sequence and used the same CNN model for training and testing also verified the robustness from the independent dataset. All the feature encoding approaches had a different impact on a single model.

In the proposed model, initially, two blocks used with the same number of layers but different values of parameters. Each block contains one convolution layer Conv1D (f, k, s) where parameter f is the number of filters, k is the kernel-size, and s represents the stride value are equal to 32, 5 and 1, respectively on both blocks. The convolution layer utilizes its ability to fetch the features by self-regulating for the input sequence of positive and negative 4mC samples. As a parameter of the convolution layer, we used L2 regularization and bias regularization to make sure that the model has no overfitting problem. We set the values for both regularizations with 0.0001 for the two Conv1D of blocks. As an activation function, an exponential linear unit (ELU) is used. Each Conv1D was followed by a group normalization layer (GN) as GroupNormalization (g) where g is a number of groups, to decrease the outcomes of convolution layers. Group normalization divides channels into groups and normalizes the feature within each group. The number of groups was set to 4 on both blocks of GN. To reduce the redundancy of the features after GN layers, we employed a max-pooling layer in each block as MaxPooling1D (l, r) where l denotes pool-size and r is the stride were set as 4 and 2, respectively. The max-pooling layer helps to reduce the dimensionality of the features from former layers. The outputs of the max-pooling layers were passed through dropout layers, Dropout (d) with a probability of 0.25 as a value of d on both blocks for the prevention of overfitting during the training. Dropout helps to switch off the effects of a few hidden nodes by adjusting the output of nodes to zero at training.

**Figure 1:**
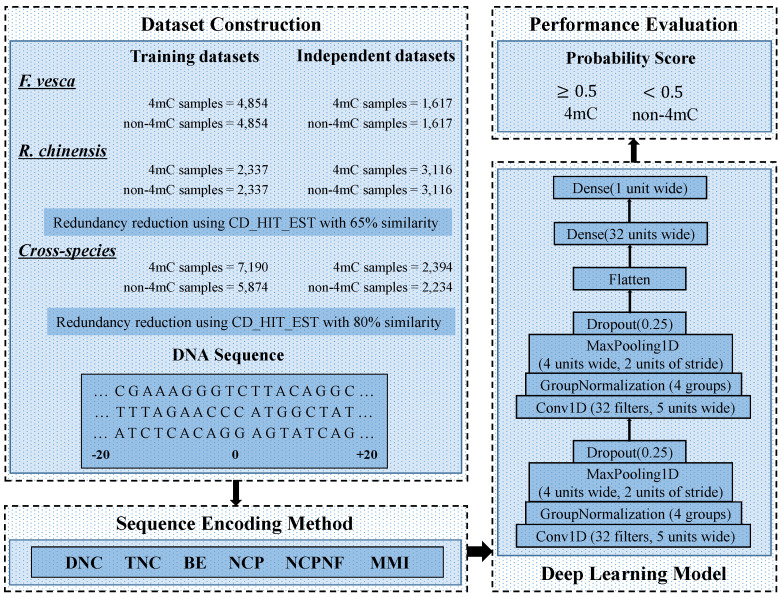
A complete structure of DNC4mC-Deep.

After both blocks, to unstack the output, a flatten function was used to squash the feature vectors from the previous layers. Right after a flatten layer, a fully connected (FC) dense layer used as Dense (n) with the number of n neurons which was set as 32 and also used the L2 regularization parameter for bias and weights with the value of 0.0001. ELU activation function used in the FC layer. At last, a FC layer was applied and used sigmoid function for the binary classification. Sigmoid is used to squeezes the values between the range of 0 and 1 to represent the probability of having 4mC and non-4mC sites. Figure 1 shows the complete architecture of the presented model.

The DNC4mC-Deep was carried out on the Keras Framework [59]. In DNC4mC-Deep we used stochastic gradient descent (SGD) optimizer with a momentum of 0.95 and the learning rate is set as 0.005. For the loss function, binary cross-entropy was used. On the fit function, we set the 100 for the epoch and 32 for the batch size. The checkpoint was used on call back function for saving the models and their best weights whereas early stopping was also implemented to halt the prediction accuracy at the time when validation stops improving. The patience level was set to 30 in early stopping.

## 4. Performance Evaluation Metrics

The performance of the prediction model can be measured by using k-fold cross-validation. In DNC4mC-Deep we used 10 fold cross-validation to achieve the foremost prediction calculation. Cross-validation is a resampling technique which provides a precise performance estimation for the predictive model. It intermixes the entire dataset and divides into a k number of clusters, where each cluster contains eight folds for training, one fold for validation, and one for testing. The model was trained and tested k times, recorded performance each time, and concised by taking the mean score for the performance evaluation. The most common criteria which is used to evaluate the performance of the predicted models are four metrics; Mathew’s correlation coefficient (MCC), accuracy (ACC), sensitivity (Sn) and specificity (Sp) with the following mathematical formulations [60,61,62].

(1) $$MCC=\frac{TP*TN-FP*FN}{\sqrt{\left(TP+FP)*(TP+FN)*(TN+FP)*(TN+FN\right)}}$$

(2) $$ACC=\frac{TP+TN}{TP+TN+FP+FN}$$

(3) $$SN=\frac{TP}{TP+FN}$$

(4) $$SP=\frac{TN}{TN+FP}$$

where TP and TN as true positive and true negative indicate the correct numbers of predicted samples for 4mCs and non-4mCs, respectively. Meanwhile, FP and FN as false positive and false negative represent the false numbers of predicted samples for 4mCs and non-4mCs, respectively. Besides, the receiver operating characteristics curve (ROC) and area under the ROC curve (AUC) were also used to show the performance of proposed model.

## 5. Results and Discussion

Six different encoding methods, namely DNC, TNC, NCP, BE, NCPNF, and MMI were used on various feature encodings for identification of the best classifier for the 4mC site prediction.

### 5.1. Performance Evaluation of Various Feature Methods on the Training Datasets

**Figure 2:**
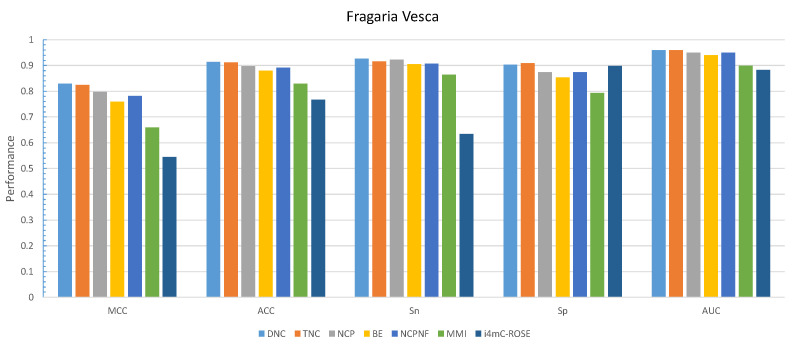
Grapical demonstration of performance comparison between six encoding methods and state-of-the-art model on training *Fragaria Vesca* dataset.

**Figure 3:**
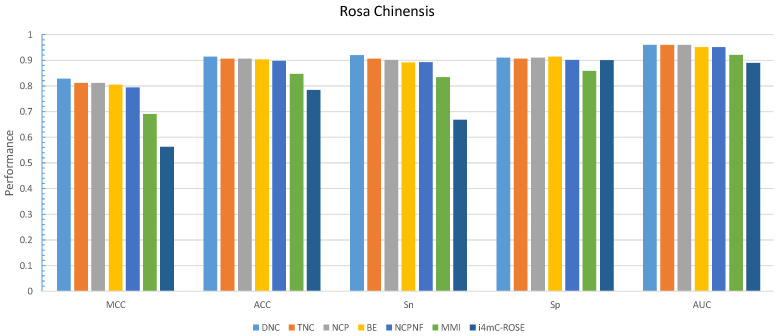
Grapical demonstration of performance comparison between six encoding methods and state-of-the-art model on training *Rosa Chinensis* dateset.

By comparing the effectiveness of the proposed methods with i4mC-ROSE model which used the same datasets, the DNC scheme yielded MCC, ACC, Sn and Sp of 0.829, 0.914, 0.926, and 0.903, respectively as the best performances for *F. vesca* dataset (Figure 2). Similarly, it achieved 0.828 for MCC, 0.914 for ACC, 0.919 for Sn and 0.910 for Sp as the maximum value on the *R. chinensis* dataset (Figure 3). The detailed performances of DNC as the best encoding method for ten different models on the R. chinensis dataset are given in Supplementary File 1. The TNC scheme yielded the highest value for Sp of 0.909 on the *F.vesca* dataset. Table 4, summarized the prediction performances by each six encoding methods and existing state-of-the-art model on *F. vesca* and *R. chinensis* datasets.

**Figure 4:**
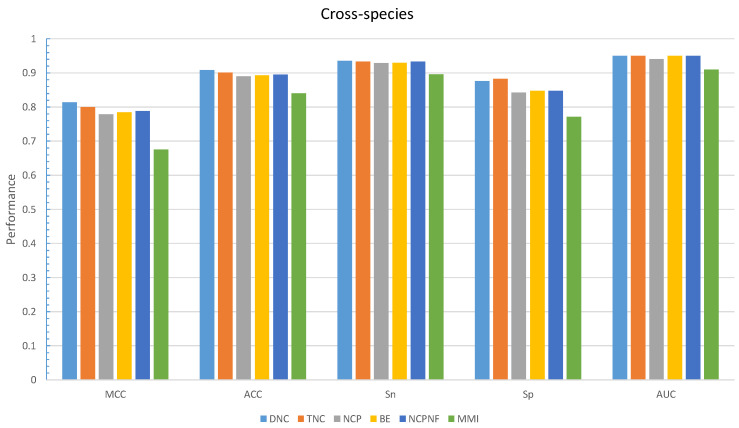
Grapical demonstration of performance comparison between six encoding methods on the training cross-species dataset.

Furthermore, the performance evaluation by six different encoding models on the cross-species dataset is shown in Figure 4. The DNC scheme yielded the highest value for all those metrics except Sp which the TNC scheme achieved the highest value of 0.882 shown in Table 5.

**Figure 5:**
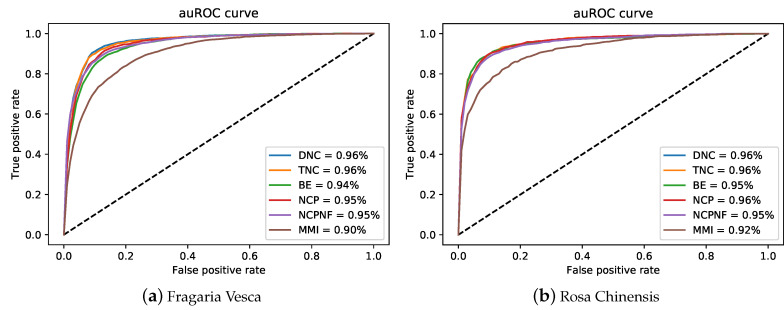
auRoc curves of six encoding methods for the proposed model on two training benchmark dataset.

**Figure 6:**
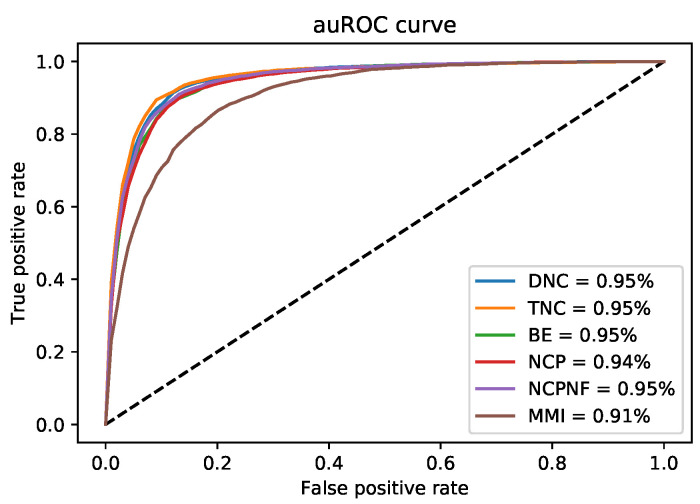
auRoc curves of six encoding methods for the proposed model on new cross-species training benchmark dataset.

The ROC curve of six encoding models was shown in Figure 5 and compared to the i4mC-ROSE model for both genomes. On the *F. vesca* dataset, the DNC and TNC achieved the best performance with an AUC value of 0.96 followed by NCP, NCPNF, BE, and MMI (Figure 5a). However, the highest AUC value was presented by DNC, TNC, and NCP of 0.96 equally and next BE, NCPNF, and MMI provided 0.95, 0.95, and 0.92, respectively on *R. chinensis* dataset (Figure 5b). Besides, DNC, TNC, BE, NCNF, and NCP all have the highest value of 0.95 on training benchmark dataset cross-species (Figure 6).

### 5.2. Performance Evaluation of Various Encoding Methods on the Independent Datasets

**Figure 7:**
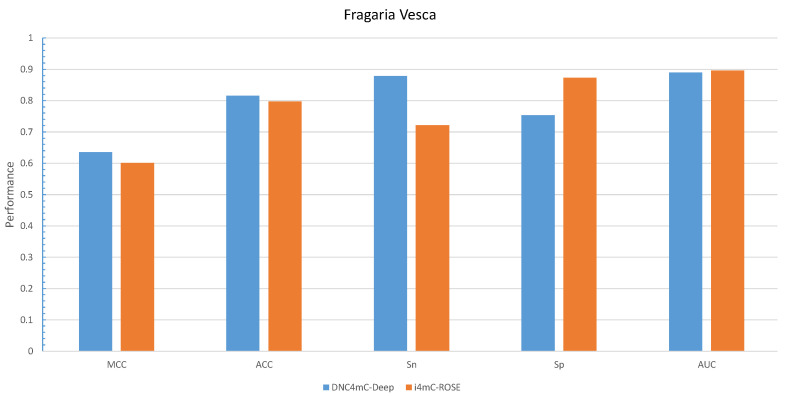
Grapical illustration of performance comparison between DNC4mC-Deep and state-of-the-art model on the independent Fragaria Vesca dataset.

**Figure 8:**
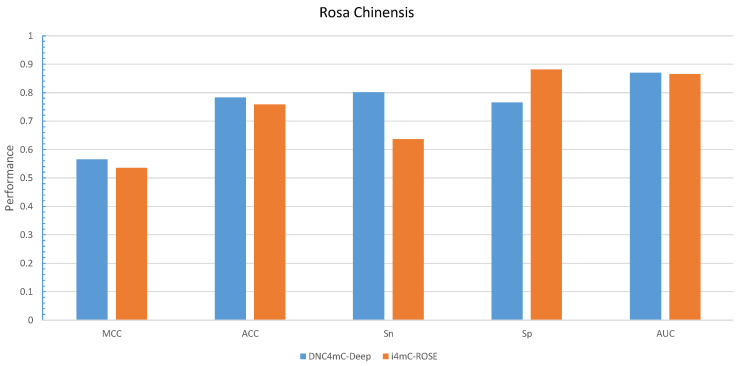
Grapical illustration of performance comparison between DNC4mC-Deep and state-of-the-art model on the independent *Rosa Chinensis* dataset.

**Figure 9:**
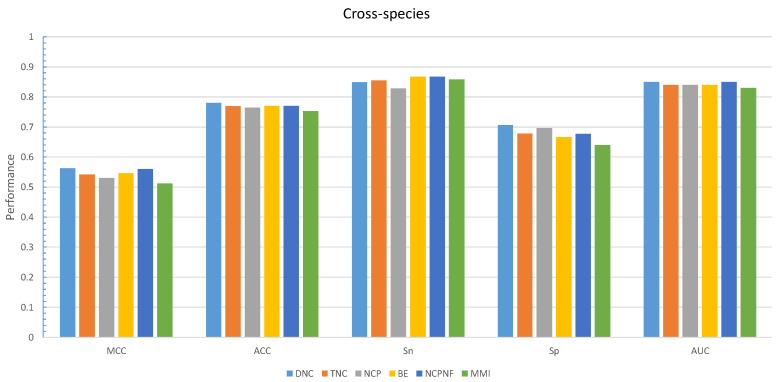
Grapical illustration of performance comparison between six encoding methods on the independent cross-species dataset.

**Figure 10:**
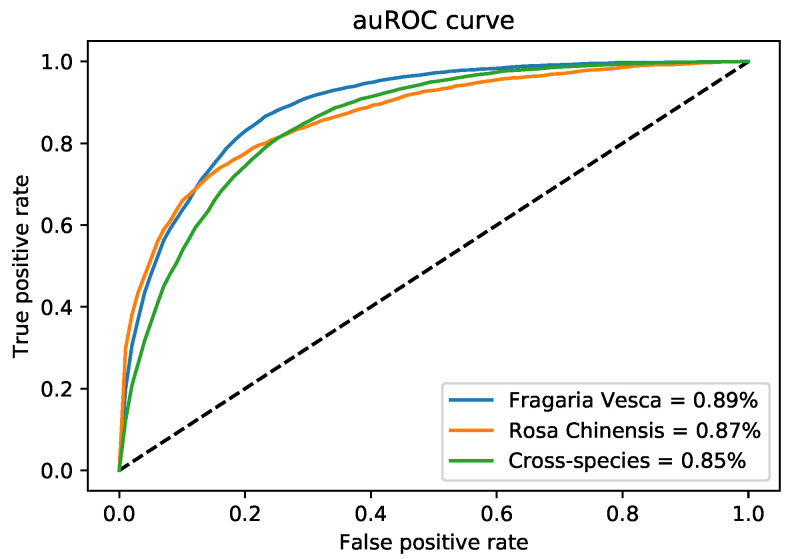
auRoc curves of three independent benchmark dataset on DNC4mC-Deep model.

We considered DNC as an encoder to characterize our proposed model, DNC4mC-Deep, due to its consistent performance on training datasets. It means we used the DNC4mC-Deep term instead of DNC scheme on the independent datasets. As represented in Table 6, the DNC4mC-Deep scheme achieved MCC, ACC, Sn and Sp of 0.635, 0.815, 0.878, and 0.753, respectively on *F. vesca* dataset (Figure 7). However, it yielded 0.565 MCC, 0.783 ACC, 0.801 Sn and 0.765 Sp on *R. chinensis* dataset (Figure 8). It can be seen clearly, comparing with the i4mC-ROSE method, the performance of the proposed predictor outperformed on both datasets. Additionally, as can be seen in Table 7, we compared the performance of six different encoding schemes on the cross-species dataset. The DNC4mC-Deep yielded the highest values for MCC, ACC, Sp, and AUC of 0.562, 0.780, 0.706, and 0.85, respectively. However, the NCPNF provided 0.871 Sn as the highest value Figure 9. Furthermore, we reached to 0.89, 0.87, and 0.85 of ROC for *F. vesca*, *R. chinensis*, and cross-species datasets which are depicted in Figure 10.

### 5.3. Interpreting Applications of Deep Learning Models

Deep learning has an ability to accomplish the state-of-the-art results but it is further challenging to construe the algorithms as a standard statistical model. In the presented work, we demonstrated two applications to understand why those deep learning models perform well apart from others and analyze their prediction by presenting the various visualization methods.

The first most authenticated and reliable method to interpret a CNN model for computational biology is silico mutagenesis which is used in several research works [35,63,64]. We computationally mutated the nucleotides by mutating each nucleotide of a single sequence with a fixed length of five nucleotides A, C, G, T, and N. During this systematic approach, the model recomposes the output of every mutation and stores the output as an absolute difference. Next, the average of mutated predictive results of the whole dataset was taken.

**Figure 11:**
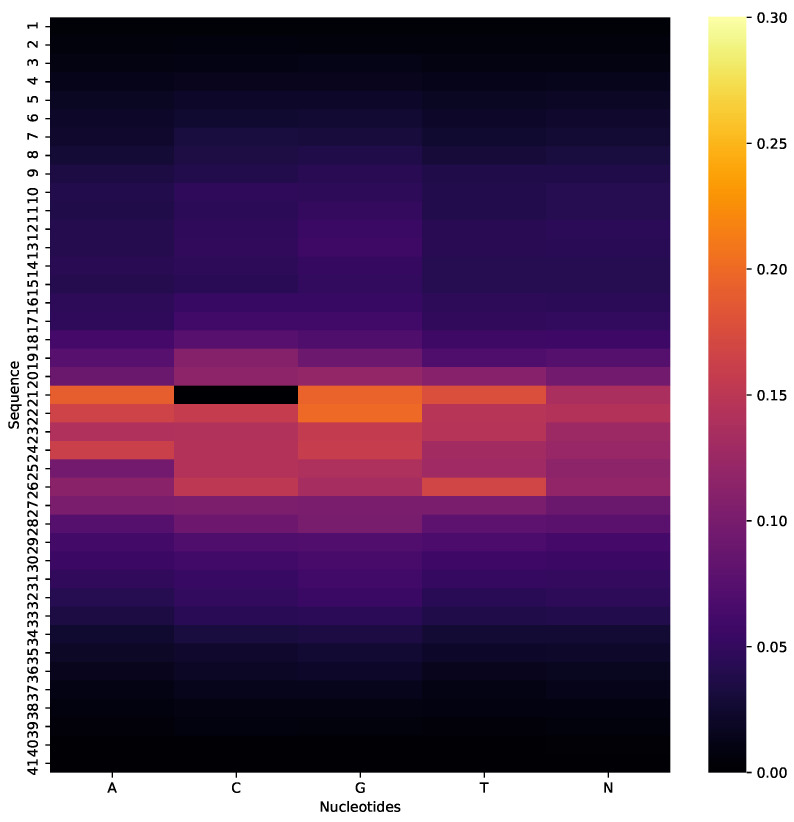
Heatmap visualization of silico mutation, where center of sequence with C nucleotide represents the highest effect on final prediction of Fragaria Vesca dataset.

A heat map was used to show the mutated modifications. CNN has the capability to visualize each convolution filter as a heat map or weight matrix. Figure 11, depicts the visualization of the mutation on *F. vesca* dataset as a local feature while learning the model. In the center of the sequences, the impact of mutation is more impactful on the final predictions because of C nucleotide which is representing the methylcytosine modification, the alteration of C nucleotide can lead to different types of gene modification. In contrast, the other sides of the heat map show the low effect of a mutation on the prediction which indicates the alteration of nucleotides cannot affect the outcome of N4-methylcytosine identification.

**Figure 12:**
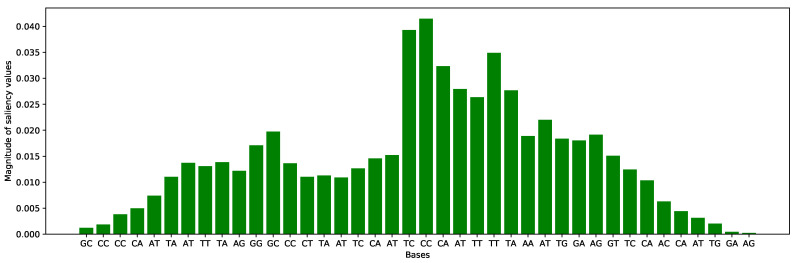
Saliency map of each di-nucleotide influence on model’s output in the Fragaria Vesca dataset.

There is another application to interpret the CNN model for knowing about the important features in the sequence, which help to gradients of the model for the final prediction. Saliency maps are the opted option to know about the most influential parts of the sequences for the classification because many researchers used in their works [36,65,66]. To visualize the effect of each position, we performed a pointwise product of the saliency map with the binary encoded sequence to acquire the derivative values for the original nucleotide letters of the sequences (A, C, G, T, and N). Samples were divided into 2-mer components across all sequences by L−k+1 formulation. In Figure 12, we can see the impact of di-nucleotide characters at each position on the output score of the whole *F. vesca* dataset. At the center of the bases, di-nucleotide motif CC has high magnitude value which represents the most important feature motif in the sequences for the prediction of the CNN model. Motif CC also indicates the N4-methycytosine modification which is our considerable problem for the prediction.

## 6. Conclusions

In this work, we presented an influential computational model named as DNC4mC-Deep to identify the N4-methylcytosine sites. There are two benchmark datasets related to the Rosaceae genome used Fragaria Vesca and Rosa Chinensis, from those two datasets we constructed a new benchmark dataset: cross-species. We used six different types of feature encoding schemes to input DNA sequence and fed to the CNN model one after another. The CNN based predictor was derived after applying the grid search algorithm. The results obtained from each encoding technique, we concluded that dinucleotide composition (DNC) outperforms and is most imperative for the strong performance of deep learning algorithms to predict 4mC sites. However, to compare with the state-of-the-art models, the CNN model with DNC encoding scheme shows the utmost effective performance and indicates the high capability of prediction. We used different evaluation metrics such as MCC, ACC, Sn, Sp, and AUC, to acquire the efficiency of the proposed predictor. Finally, we interpreted our deep learning model from two techniques: silico mutagenesis and saliency map. DNC4mC-Deep can make a high impact on the biologist to identify the N4-methylcytosine sites and can be used in brain development abnormalities. In the future, we will extend the work to prepare some new datasets and make computational models related to deep learning. Meanwhile, we established the webserver http://home.jbnu.ac.kr/NSCL/DNC4mC-Deep.htm, for users to achieve their desired results easily.

## Figures and Tables

**Table 1:** Benchmark datasets demonstration.

Species	Dataset	Training Dataset	Total	Independent Dataset	Total
*F. vesca*	4mC samples	4854	9708	1617	3234
	non-4mC samples	4854		1617	
*R. chinensis*	4mC samples	2337	4674	779	1558
	non-4mC samples	2337		779	
*Cross-species*	4mC sampels	7190	13,064	2394	4628
	non-4mC samples	5874		2234	

**Table 2:** Cluster of nucleotides based on chemical properties.

Chemical Property	Class	Nucleotides
Ring structure	Two ring	A, G
	One ring	C, T
Hydrogen bond	Strong	C, G
	Weak	A, T
Functional group	Amino	A, C
	Keto	G, T

**Table 3:** Hyper-parameters tuning demonstration.

Parameters	Range
Convolution layers	[1, 2, 3, 4, 5]
Filters in convolution Layer	[8, 12, 16, 22, 32, 42, 64, 128]
Filter size	[2, 3, 4, 5, 6, 7, 8, 10, 12, 14]
Pool-size in Maxpooling	[2, 4]
Stride length in Maxpooling	[2, 4]
Dropout values	[0.2, 0.25, 0.3, 0.35, 0.4]

**Table 4:** Performance evaluation of six encoding methods with state-of-the-art model on training benchmark dataset for *F. vesca* and *R. chinensis* species.

Dataset	Method	MCC	ACC	Sn	Sp	AUC
Fragaria Vesca	DNC	0.829	0.914	0.926	0.903	0.96
	TNC	0.825	0.912	0.916	0.909	0.96
	NCP	0.797	0.898	0.922	0.874	0.95
	BE	0.760	0.879	0.905	0.854	094
	NCPNF	0.782	0.891	0.907	0.874	0.95
	MMI	0.659	0.829	0.864	0.794	0.90
	i4mC-ROSE	0.545	0.767	0.635	0.899	0.88
Rosa Chinensis	DNC	0.828	0.914	0.919	0.910	0.96
	TNC	0.811	0.906	0.906	0.906	0.96
	NCP	0.811	0.906	0.901	0.910	0.96
	BE	0.805	0.903	0.891	0.914	0.95
	NCPNF	0.794	0.897	0.892	0.901	0.95
	MMI	0.691	0.846	0.833	0.858	0.92
	i4mC-ROSE	0.563	0.784	0.668	0.900	0.89

**Table 5:** Performance evaluation of six encoding methods on training benchmark dataset for cross-species.

Dataset	Method	MCC	ACC	Sn	Sp	AUC
Cross-species	DNC	0.814	0.908	0.935	0.876	0.95
	TNC	0.800	0.901	0.933	0.882	0.95
	NCP	0.779	0.890	0.929	0.843	0.94
	BE	0.785	0.893	0.930	0.848	0.95
	NCPNF	0.788	0.895	0.933	0.848	0.95
	MMI	0.676	0.840	0.896	0.772	0.91

**Table 6:** Performance evaluation between the DNC4mC-Deep and state-of-the-art model on independent benchmark dataset for *F. vesca* and *R. chinensis* species.

Dataset	Method	MCC	ACC	Sn	Sp	AUC
Fragaria Vesca	DNC4mC-Deep	0.635	0.815	0.878	0.753	0.89
	i4mC-ROSE	0.601	0.797	0.721	0.873	0.89
Rosa Chinensis	DNC4mC-Deep	0.565	0.783	0.801	0.765	0.87
	i4mC-ROSE	0.535	0.759	0.636	0.881	0.86

**Table 7:** Performance evaluation of DNC4mC-Deep and other five encoding methods on independent benchmark dataset for cross-species.

Dataset	Method	MCC	ACC	Sn	Sp	AUC
Cross-species	DNC4mC-Deep	0.562	0.780	0.849	0.706	0.85
	TNC	0.542	0.769	0.854	0.678	0.84
	NCP	0.530	0.764	0.828	0.696	0.84
	BE	0.546	0.770	0.867	0.666	0.84
	NCPNF	0.560	0.777	0.871	0.677	0.85
	MMI	0.512	0.753	0.858	0.640	0.83

## Supplementary Materials

The following are available online at https://www.mdpi.com/2073-4409/9/8/1756/s1. Supplementary File 1: The detailed performances of ten different models on DNC.
