# Supplementary material for: DNC4mC-Deep: Identification and Analysis of DNA N4-Methylcytosine Sites Based on Different Encoding Schemes By Using Deep Learning
Source: Cells. 2020 Jul 22;9(8):1756. doi: 10.3390/cells9081756 (PMC7465362; doi:10.3390/cells9081756)
Supplement: Supplementary file 1 [file cells-09-01756-s001.pdf]

Supplementary material

The results of 10 different models using the DNC encoding method are given below. The architecture of each model and its results based on four standard evaluation metrics are shown.

Model 1 Results:

Conv1D (8, 2), Maxpooling (2, 2), Dropout (0.2), Dense (16), Dense (1).

MCC	ACC	Sn	Sp	AUC
70.61	85.30	85.75	84.86	0.92

Model 2 Results:

Conv1D (8, 4), Maxpooling (2, 2), Dropout (0.2), Dense (16), Dense (1).

MCC	ACC	Sn	Sp	AUC
74.49	87.25	87.04	87.45	0.93

Model 3 Results:

Conv1D (16, 5), Maxpooling (4, 2), Dropout (0.2), Dense (16), Dense (1).

MCC	ACC	Sn	Sp	AUC
74.68	87.34	87.26	87.42	0.93

Model 4 Results:

Conv1D (8, 4), Maxpooling (2, 2), Dropout (0.2), Conv1D (16, 5), Maxpooling (2, 2), Dropout (0.25), Dense (16), Dense (1).

MCC	ACC	Sn	Sp	AUC
68.46	84.23	84.45	84.01	0.92

Model 5 Results:

Conv1D (16, 6), BatchNormalization (), Maxpooling (4, 2), Dropout (0.25), Conv1D (35, 8), BatchNormalization (), Maxpooling (4, 2), Dropout (0.30), Dense (16), Dense (1).

MCC	ACC	Sn	Sp	AUC
72.36	86.71	86.85	85.50	0.92

Model 6 Results:

Conv1D (64, 12), Maxpooling (4, 2), Dropout (0.35), Conv1D (42, 10), Maxpooling (4, 2), Dropout (0.35), Dense (32), Dense (1).

MCC	ACC	Sn	Sp	AUC
75.81	87.90	88.36	87.45	0.94

Model 7 Results:

Conv1D (8, 4), Maxpooling (2, 2), Dropout (0.2), Conv1D (16, 5), Maxpooling (2, 2), Dropout (0.2), Conv1D (32, 6), Maxpooling (2, 2), Dropout (0.3), Dense (8), Dense (1).

MCC	ACC	Sn	Sp	AUC
65.99	82.96	85.13	80.81	0.90

Model 8 Results:

Conv1D (16, 3), Maxpooling (2, 2), Dropout (0.25), Conv1D (32, 5), Maxpooling (2, 2), Dropout (0.3), Conv1D (22, 4), Maxpooling (4, 2), Dropout (0.4), Dense (16), Dense (1).

MCC	ACC	Sn	Sp	AUC
66.34	83.17	82.97	83.37	0.90

Model 9 Results:

Conv1D (64, 12), Maxpooling (4, 2), Dropout (0.4), Conv1D (64, 8), Dropout (0.3), Conv1D (32, 5), Maxpooling (2, 2), Dropout (0.3), Dense (32), Dense (1).

MCC	ACC	Sn	Sp	AUC
74.52	87.26	87.07	87.45	0.94

Model 10 Results:

Conv1D (128, 14), GroupNormalization (4), Maxpooling (2, 2), Dropout (0.4), Conv1D (64, 8), GroupNormalization (4), Dropout (0.3), Conv1D (32, 5), GroupNormalization (4), Maxpooling (2, 2), Dropout (0.3), Conv1D (16, 4), GroupNormalization (4), Maxpooling (2, 2), Dropout (0.35), Dense (32), Dense (16), Dense (1).

MCC	ACC	Sn	Sp	AUC
75.35	87.67	88.12	87.23	0.94
